# Pregabalin beyond pain: A neuroprotective alternative in peripheral nerve injury

**DOI:** 10.1016/j.clinsp.2026.100957

**Published:** 2026-07-17

**Authors:** Antonio Alcántara Montero

**Affiliations:** Centro de Salud Trujillo, Trujillo (Cáceres), Spain

The experimental study by Ateş and colleagues provides novel evidence on the neuroprotective potential of pregabalin compared with methylprednisolone in a rat axonotmesis model. The authors demonstrate that pregabalin, particularly at higher doses, exerts regenerative and functional effects similar to those of steroids, and the combination of both treatments yields superior outcomes.^1^ This finding is relevant, as the search for “steroid-sparing” strategies in peripheral nerve injuries remains a pressing clinical need.

The study presents several strengths: a solid experimental design with prior sample size calculation, the inclusion of multiple comparison groups, and a multimodal evaluation encompassing functional parameters such as the Sciatic Functional Index, electrophysiological measures through electromyography, histopathological analysis, immunohistochemistry, and biochemical markers. The consistency of results across these dimensions reinforces the conclusion that pregabalin may contribute to nerve regeneration beyond its well-known analgesic role.

Nevertheless, some limitations should be noted. The follow-up was limited to 28-days, a relatively short period to assess complete nerve regeneration, which often extends over several months.^2^ In addition, behavioral measures of neuropathic pain (such as allodynia or hyperalgesia) were not included, preventing assessment of whether the analgesic action of pregabalin integrates with its neuroprotective potential. This aspect is crucial, given that pregabalin is well established in the treatment of neuropathic pain^3^ and fibromyalgia.^4^ Furthermore, the methylprednisolone regimen employed (1 mg/kg orally) may not reflect routine clinical practice, where doses and routes differ and have been primarily studied in spinal cord injury.^5^ These considerations should temper extrapolation to human settings. The axonotmesis model itself has translational constraints, including species differences and limited correlation with long-term human outcomes.

Pregabalin’s binding to the α2δ subunit of voltage-gated calcium channels reduces excitatory neurotransmitter release, which may attenuate excitotoxicity and neuroinflammation. This mechanism provides biological plausibility for both analgesic and potential neuroprotective effects, though distinguishing symptomatic modulation from structural repair remains essential.

The clinical implications of this work are significant but must be framed cautiously. Rather than immediate recommendations, these findings generate hypotheses for future translational studies. The possibility of using pregabalin as a neuroprotective agent opens a new therapeutic horizon in peripheral nerve injuries, where pharmacological options are limited, and surgical outcomes do not always guarantee full functional recovery.^6^ The combination of pregabalin and steroids could allow reduction of corticosteroid doses, minimizing adverse effects without compromising neuroprotective efficacy. This approach aligns with current trends in neurology and pain medicine to minimize steroid exposure, whose side effects are well known.^7^^,^^8^ Finally, the dual potential of pregabalin ‒ analgesic and neuroprotective ‒ could favor a more comprehensive recovery, addressing both nerve regeneration and neuropathic pain control, which is one of the main causes of disability after peripheral nerve injuries.^9^

Historically, methylprednisolone became the reference treatment for acute spinal cord injury following the National Acute Spinal Cord Injury Studies (NASCIS).^5^ However, its use has been debated due to adverse effects and the lack of sustained benefit in some patients. In the peripheral nervous system, corticosteroids have been employed to reduce edema and inflammation, but they have never been consolidated as a standard therapy for regeneration. In this context, the search for pharmacological alternatives with a better safety profile is a priority. Pregabalin, widely used in neuropathic pain, offers a distinct mechanism of action: modulation of calcium channels and reduction of excitatory neurotransmitter release. Other candidate strategies, such as antioxidants, neurotrophic factors, or conditioning lesions, have also been explored, but none have yet achieved consistent clinical translation.

From a translational perspective, the findings of Ateş et al. suggest several lines of investigation. In surgical specialties where peripheral nerve injuries are frequent, the incorporation of pregabalin as an adjuvant treatment could improve functional recovery. In pain medicine, its combined use with steroids could reduce the need for high corticosteroid doses, decreasing metabolic and immunological complications. In rehabilitation, the integration of pregabalin into multimodal protocols could facilitate motor and sensory recovery, improving patients’ quality of life. These scenarios should be considered hypotheses for clinical trials rather than current practice recommendations.

Moreover, this study invites reflection on the role of neurotrophic factors and the inflammatory response in peripheral regeneration. The demonstration that pregabalin may indirectly modulate processes such as Nerve Growth Factor release or Nuclear Factor kappa B expression suggests that its benefit could extend beyond symptomatic pain control. The interaction between pharmacotherapy and cellular repair mechanisms, such as Schwann cell proliferation and myelin clearance, is a fertile field for future research.^10^

It is also important to consider the clinical heterogeneity of peripheral nerve injuries. Not all lesions present the same degree of sensory, motor, or autonomic involvement, and the response to pharmacological treatments may vary depending on the extent of damage and the time elapsed before intervention. Pregabalin, due to its safety and tolerability profile, could be particularly useful in patients with comorbidities that limit corticosteroid use, such as diabetes, hypertension, or immune disorders. This reinforces the need for clinical trials including diverse and representative populations. Longer follow-up and clinically relevant dosing regimens will be essential to validate these hypotheses.

Finally, the study by Ateş et al. contributes to a broader debate on the integration of pharmacological and surgical therapies in the management of nerve injuries. Peripheral regeneration is a complex process that depends on both biological factors and technical interventions. The possibility of enhancing repair through agents such as pregabalin opens the door to combined strategies that could optimize functional outcomes and reduce the burden of disability associated with these injuries.

Future studies should extend follow-up, incorporate neuropathic pain measures, and explore dosing regimens closer to clinical practice. Well-designed clinical trials will be essential to confirm whether the promising experimental findings translate into patient benefit.

In conclusion, Ateş et al. provide compelling experimental evidence that pregabalin may represent an alternative or adjunct to methylprednisolone in peripheral nerve regeneration. These findings should be interpreted as hypothesis-generating and warrant rigorous translational and clinical validation. Their work opens a line of research that deserves exploration, with the potential to inform future therapeutic strategies in this field.

## Funding

No funding was received for this manuscript.

## Data availability

The datasets generated and/or analyzed during the current study are available from the corresponding author upon reasonable request.

## Conflict of interest

The author declares no conflicts of interest.
